# Combination of Strobilurin and Triazole Chemicals for the Management of Blast Disease in Mushk Budji -Aromatic Rice

**DOI:** 10.3390/jof7121060

**Published:** 2021-12-10

**Authors:** Fayaz Ahmad Mohiddin, Nazir A. Bhat, Shabir H. Wani, Arif H. Bhat, Mohammad Ashraf Ahanger, Asif B. Shikari, Najeebul Rehman Sofi, Shugufta Parveen, Gazala H. Khan, Zaffar Bashir, Pavla Vachova, Sabry Hassan, Ayman EL Sabagh

**Affiliations:** 1 Mountain Research Centre for Field Crops (MRCFC)-Khudwani, Molecular Plant Pathology Laboratory, SKUAST-Kashmir, Srinagar 192231, India; arifsayar21@gmail.com; 2 MRCFC-Khudwani, SKUAST-Kashmir, Srinagar 192231, India; nazirpathology@gmail.com (N.A.B.); shugufta1351@gmail.com (S.P.); 3 MCRS, Sagam, SKUAST-Kashmir, Srinagar 190025, India; mashrafjs@gmail.com; 4 Rice Genomics Laboratory, MRCFC-Khudwani, SKUAST-Kashmir, Srinagar 192231, India; asifshikari@gmail.com (A.B.S.); najeeb_sofi@rediffmail.com (N.R.S.); gazalakhan5818@gmail.com (G.H.K.); 5 Department of Microbiology, University of Kashmir, Srinagar 190006, India; zaffarsahib@gmail.com; 6 Department of Botany and Plant Physiology, Faculty of Agrobiology, Food and Natural Resources, Czech University of Life Sciences, 165 00 Prague, Czech Republic; vachovap@af.czu.cz; 7 Department of Biology, College of Science, Taif University, P.O. Box 11099, Taif 21944, Saudi Arabia; hassan@tu.edu.sa; 8 Department of Agronomy, Faculty of Agriculture, Kafrelsheikh University, Kafr El-Shaikh 33516, Egypt; aymanelsabagh@gmail.com

**Keywords:** rice, leaf blast, neck blast, management, fungicides

## Abstract

Rice blast is considered one of the most important fungal diseases of rice. Although diseases can be managed by using resistant cultivars, the blast pathogen has successfully overcome the single gene resistance in a short period and rendered several varieties susceptible to blast which were otherwise intended to be resistant. As such, chemical control is still the most efficient method of disease control for reducing the losses caused due to diseases. Field experiments were conducted over two successive years, 2018 and 2019, in temperate rice growing areas in northern India. All the fungicides effectively reduced leaf blast incidence and intensity, and neck blast incidence under field conditions. Tricyclazole proved most effective against rice blast and recorded a leaf blast incidence of only 8.41%. Among the combinations of fungicides, azoxystrobin + difenoconazole and azoxystrobin + tebuconazole were highly effective, recording a leaf blast incidence of 9.19 and 10.40%, respectively. The chemical combination mancozeb + carbendazim proved less effective in controlling the blast and it recorded a disease incidence of 27.61%. A similar trend was followed in neck blast incidence with tricyclazole, azoxystrobin + difenoconazole, and azoxystrobin + tebuconazole showing the highest levels of blast reductions. It is evident from the current study that the tested fungicide combinations can be used as alternatives to tricyclazole which is facing the challenges of fungicide resistance development and other environmental concerns and has been banned from use in India and other countries. The manuscript may provide a guideline of fungicide application to farmers cultivating susceptible varieties of rice.

## 1. Introduction

**Figure 1:**
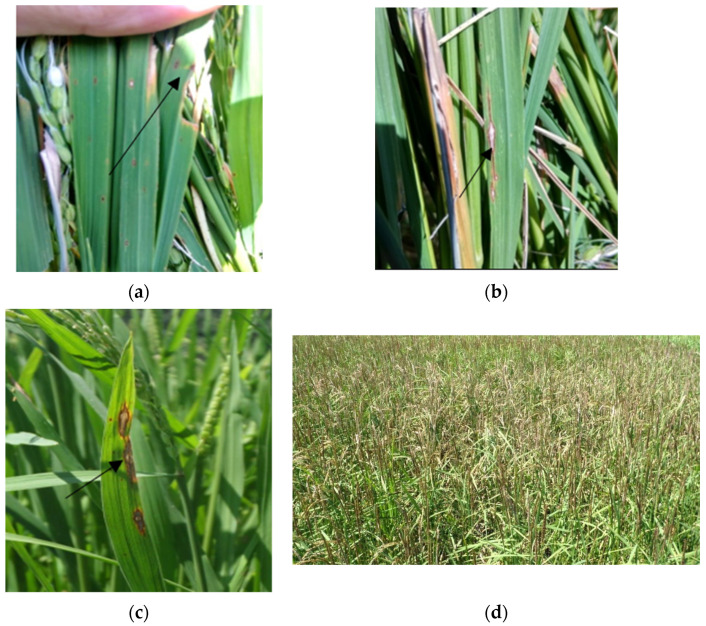
Rice blast symptoms in the field: (**a**) initial appearance of blast lesions (arrow); (**b**) characteristic diamond-shaped blast lesions showing vertical extension (arrow); (**c**) coalescing of blast lesions; (**d**) rice field of Mushk Budji cultivar heavily infested with rice blast. (Source: photographs are from our laboratory).

With the growing world population, food security and crop protection have become highly important. Rice meets the daily food requirements of more than 3.5 billion people [1]. India ranks second in rice production after China [2] and is the largest producer and exporter of aromatic Basmati rice in the world [3]. Rice blast, initiated by *Pyricularia oryzae*, is the major impediment in world rice production and inflicts heavy yield losses [4]. Cases of rice blast have been reported from more than 85 countries and it inflicts devastating crop losses. It is the most critical disease of cultivated rice around the world and can cause 100% crop losses if adequate management measures are not adopted [5]. Currently, the estimated declines in world rice production of about 30% are caused due to rice blast and only these losses if prevented would be enough to feed more than 60 million people [6,7]. The blast fungus is capable of infecting rice at any stage of the host life cycle. The disease appears early as white to grey/brown leaf spots or lesions (Figure 1), followed by nodal rot and as neck blast, which can cause necrosis and frequently breakage of the host panicles [8]. Cultivation of resistant varieties, fungicide applications, and manipulating planting dates, fertilizer applications, and irrigation are the frequently used approaches for rice blast management [9,10,11,12]. Genetic resistance to the rice blast pathogen seems to be an ecofriendly and effective management strategy, but the rice blast fungus has been reported to be rapidly overcoming this resistance [13,14]. Current low-cost protection strategies include the planting of uninfected seeds, limiting nitrogen fertilizers, perpetual field flooding, and post-harvest burning of plant remains [15]; however, these measures are rendered ineffective once infection is established in the field. Seed treatments with systemic fungicides and foliar sprays have remained effective from the beginning in rice blast management [16,17,18,19,20]. The current major strategies for managing the disease are the development of resistant varieties and application of fungicides [21]. At present, the blast disease is mainly managed by cultivating the resistant varieties; however, this strategy is often challenged by the development of new pathogenic races resulting in the resistance breakdown within a few years. It has also been reported that sometimes farmers prefer to grow susceptible rather than resistant varieties because of high consumer demand. In such cases, the disease in susceptible rice varieties is managed by the application of chemical fungicides [22]. Hence, chemical control is still widely practiced and is the most successful strategy for managing crop losses due to blast globally [21,23]. The fungicides chlorothalonil, tricyclazole, hexaconazole, carbendazim, and propiconazole have been reported to be effective in the management of rice blast disease [24].

Several fungicides belonging to different groups have been synthesized and evaluated for use in the rice ecosystem throughout the world. More than 30 fungicides with recommended concentrations have been registered for use in rice and several new molecules are undergoing testing [25]. In India, a number of chemicals and various schedules for spraying have been recommended on the basis of work done in the past several decades [26]. However, the continuous use of the same chemicals year after year results in the development of fungicide resistance. Tricyclazole was the most effective fungicide among all the chemicals in rice blast management and has played a big role in successful rice cultivation for decades particularly in India. However, due to increasing concerns of its hazardous effects on human health, it has now been banned from use in India. Hence, a need arises to find the suitable alternative to tricyclazole in blast management as well as to address the problem of fungicide resistance in the fungal population of the blast pathogen. It has been reported that triazole and strobilurin together have significant effects against rice blast and the fungicides viz., fluopyram + tebuconazole, difenconazole + propiconazole, flutriafole, and azoxystrobin achieved stronger fungicidal activity against rice blast diseases at a recommended concentration mostly found safe throughout the world [27]. Fungicidal control is largely practiced for blast disease in temperate or subtropical rice cultivation, mainly in Japan, China, South Korea, Taiwan and, increasingly, Vietnam. The majority of the fungicides used in blast control are protectants. In early years, copper and mercury compounds were recommended against blast but were found not suitable because of phytotoxicity and mammalian toxicity. Current major products are mainly systemics with a residual activity of at least 15 days, although older organophosphorous products such as edifenphos are still widely used. The modern rice fungicides include isoprothiolane, probenazole, pyroquilon, and tricyclazole [28,29] and are applied as foliar sprays, as granules into water or seed-box treatments (irrigated lowland rice), or as seed dressings for upland rice. In recent years, newer melanin biosynthesis inhibitors such as carpropamid [30] or broad-spectrum fungicides like azoxystrobin (strobilurin) [31] have gained favor.

Various workers have suggested the use of fungicides belonging to different groups in rotation to avoid the development of resistance in pathogen populations [32]. No systematic studies have been undertaken so far on the evaluation of new combination fungicides for blast management in susceptible aromatic rice (Mushk Budji). Keeping in view the increasing demand of Mushk Budji rice in the local markets as well as its huge export potential and the challenge of blast disease management, the aim of our study was to evaluate and screen some new combination fungicides for the management of rice blast disease under temperate Indian conditions.

## 2. Materials and Methods

### 2.1. Characterization of Blast Pathogen

#### 2.1.1. Isolation

**Figure 2:**
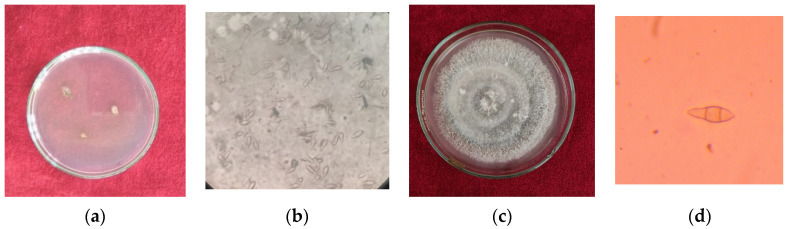
Cultural and morphological characterization of *Pyricularia oryzae* (fungus) causing blast disease of rice (**a**). Initial growth of fungus on PDA (**b**). Various spores of fungus (**c**). Growth of fungus after 20 days on culture plate (**d**) Single spore of fungus (Source: photographs are from our laboratory).

The leaves exhibiting typical blast symptoms were used for isolation of the pathogen. The infected leaves were collected from the field and examined under a microscope for the associated pathogen. The isolation was completed by the tissue bit method [33]. The symptomatic leaves were cut into small pieces of 2–3 cm with a sterilized blade. The bits were cut in such a manner that each bit contained a portion of healthy tissue along with the infected portion. Surface sterilization of the bits was conducted by a 0.1 percent mercuric chloride solution for 30 s followed by thrice rinsing with distilled sterilized water to remove the last trace of the mercuric chloride solution. The bits were then dried in blotter paper and transferred to a potato dextrose agar medium and incubated at 25 ± 1 °C. The plates were observed regularly until mycelial growth occurred which was then subcultured in the PDA slants for maintenance at 4 °C (Figure 2).

#### 2.1.2. Morphological Characteristics

The morphological and cultural studies of the pathogen were conducted under laboratory conditions from the culture growth on PDA for 15–25 days at 25 ± 1 °C. Observations of different morphological characters viz, mycelium, conidia, and colony characteristics were studied. The 3 mm disc from pure culture was inoculated on a petri plate containing PDA. Visual growth of the fungus was observed after 15 days. The color of the colony varied from whitish grey to blackish grey with raised whitish mycelium. The margins were smooth, having an average diameter of 51 mm (Figure 2). Mycelium of the isolated fungus had a breadth of 4.76 µ. The conidia were pyriform, measuring 22.69 µ × 9.02 µ, septate, having 2 septa (Figure 2). The colony characteristics as well as size and shape of spores are important factors for fungal identification and are in agreement with those described by [34,35,36].

#### 2.1.3. Field Experiments

Field experiments were conducted on susceptible indigenous rice cultivar “Mushk Budji” under natural epiphytotic conditions at the Mountain Research Centre for Field Crops (MRCFC), Khudwani (33°70′ N, 75°10′ E), Anantnag district of the union territory of Jammu and Kashmir in northern India. MRCFC, Khudwani is considered a hot spot for rice blast disease and is the lead center of rice blast research in India.

The experiment was laid out in a randomized complete block design with three replications for each treatment (30 plots, 10 treatments, 5 rows in each plot, 1 hill) during kharif season in 2018 and 2019. The seeds procured from MRCFC, Khudwani were sown in May and transplanting was performed in the month of June in both the years. The plots were 4 m × 4 m in size separated by 1 m. Thirty-days-old seedlings developed at MRCFC Khudwani were transplanted at 20 × 10 cm spacing in the plots and conventional cultivation practices were followed. The experimental fields were naturally infested with the blast pathogen.

The sprayer used was the 3WBS-16A2 electric air-pressure knapsack sprayer equipped with twin hollow cone nozzles. The tank capacity was 16 L. The sprayer had a pressure pump which provided a maximum pressure of 4 bars and a flow rate of 1.6 L/min. The length of the lance of the sprayer was 81 cm and the spray swath width was approximately 2.5 m. The traveling speed was approximately 1.0–1.5 km/h generating a spray volume of about 300 L/ha. The spray was completed in a swinging spraying pattern and the working height of the nozzle was 0.5 m above the crop canopy. The experiment consisted of 10 treatments including 9 fungicides and one negative control (water spray). The fungicides included: melanin biosynthesis inhibitors—reductase (Force 11^TM^, Insecticides Ltd., Delhi, India); combination fungicides with sterol biosynthesis and QoI inhibitors (triazole plus strobilurin fungicides—Amistar^®^ Top, Syngenta India Ltd., Pune, Maharashtra, India; Custodia, ADAMA India, Hyderabad, India; Nativo^®^Bayer Cropscience Ltd., Thane, Maharashtra, India); combination fungicides with succinate dehydrogenase inhibitors and sterol biosynthesis inhibitors (pyrazole-carboximide plus triazole fungicide- Adexar^®^, BASF, Bandra East, MumbaiIndia); combination fungicides with sterol biosynthesis inhibitors + β-tubulin polymerization inhibitors (triazole plus benzimidazoles fungicide-Lustre, Dhanuka Agritech, New Delhi, India); combination fungicides with sterol biosynthesis inhibitors + enzyme system inhibitors (triazole plus dithiocarbamate fungicide- Merger, Indofil Industries Ltd., Mumbia, Maharashtra, India; Avatar, Indofil Industries Ltd., Mumbia, Maharashtra, India); and combination fungicides with enzyme system inhibitors + β-tubulin polymerization inhibitors (dithiocarbamate plus benzimidazole fungicide- Sprint^®^, Indofil Industries Ltd., Mumbia, Maharashtra, India).

Three sprays were performed at weekly intervals with the first spray at the booting stage, with separate portable knapsack sprayers in July and August for two consecutive years, 2018 and 2019. The aim of the fungicidal sprays was to control the early season leaf blast and late season neck blast. The data on leaf blast incidence and intensity were recorded one week after the last spray; however, neck blast incidence was recorded one week before harvesting the crop. From each plot, 25 hills were randomly chosen, and all the tillers were observed for the presence of disease. The percent of leaf blast incidence and neck blast incidence were calculated by using the formula:

$$\mathrm{Percent}\;\mathrm{disease}\;\mathrm{incidence}=\frac{\mathrm{No}.\;\mathrm{of}\;\mathrm{diseased}\;\mathrm{plants}}{\mathrm{Total}\;\mathrm{No}.\;\mathrm{of}\;\mathrm{plants}\;\mathrm{observed}}\times 100$$

The leaf blast severity was recorded by randomly selecting 25 plants per plot, 7 days after the last spray, and adopting the 0–9 disease rating scale of the International Rice Research Institute [37]; (Table 1) based on the percent of plant tissue affected.

The leaf blast severity was worked out as per [38]:

$$\mathrm{Severity}=\frac{\sum \left(n\times v\right)\times 100}{N\times G}$$

where n = number of infected leaves in a category; v = category value (0–9); N = total number of leaves observed in each replication; and G = highest category value.

The percent of disease control for leaf and neck blast was worked out using the following formula:

$$\mathrm{PDC}=\frac{C-T}{C}\times 100$$

where PDC is percent of disease control, C is the disease incidence in control plot, and T is the disease incidence in fungicide treated plots.

The data on yield parameters were recorded at crop maturity. A 3 × 2 m area was marked in each plot with the help of a wire frame as per [39]. All the tillers within this area were harvested for the estimation of the yield.

### 2.2. Statistical Analysis

The data on rice blast incidence, severity, and yield were analyzed with the statistical program SPSS v.19 and means were compared with Duncan’s multiple range test (DMRT) at *p* < 0.05 for differences due to different fungicide treatments.

## 3. Results

### 3.1. Effect of Fungicide Treatments on Leaf Blast Incidence

**Figure 3:**
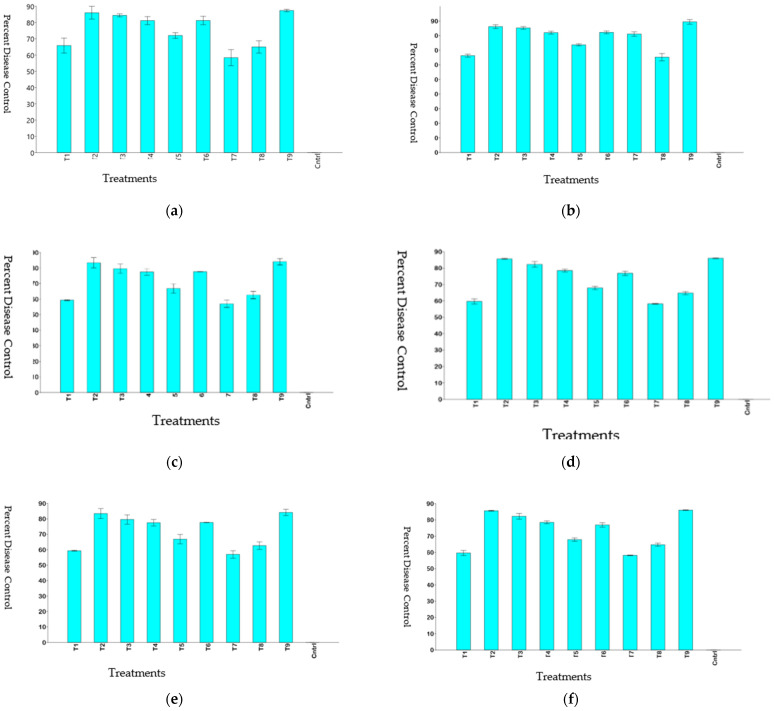
Percent of disease control to leaf blast incidence: (**a**) 2018; (**b**) 2019, leaf blast severity; (**c**) 2018; (**d**) 2019 and nodal blast incidence; (**e**) 2018; (**f**) 2019. Bars correspond to the retransformed means and the upper and lower 95% confidence intervals of the mean are shown as error bars.T1 = flusilazole + carbendazim, T2 = azoxystrobin+ difenoconazole, T3 = azoxystrobin + tebuconazole, T4 = tricyclazole + mancozeb, T5 = zineb + hexaconazole, T6 = trifloxystrobin + tebuconazole, T7 = mancozeb + carbendazim, T8 = fluxapyroxad + epoxiconazole, T9 = tricyclazole, and T10 = Control (Cntrl).

It is evident from the data that all the fungicides were highly effective in checking blast incidence. The fungicides azoxystrobin+ difenoconazole and azoxystrobin + tebuconazole were found most effective in reducing leaf blast incidence in both the years. In 2018, they showed a slightly activity as compared to tricyclazole but in 2019, their efficiency was at par with tricyclazole (*p* < 0.05). These fungicides recorded maximum percent disease control when compared to all other combination fungicides (Figure 3a,b). These combination fungicides were followed by trifloxystrobin + tebuconazole and tricyclazole + mancozeb which also recorded a significant reduction in leaf blast incidence in both the years 2018 and 2019. The fungicide combinations zineb + hexaconazole, flusilazole + carbendazim, and fluxapyroxad + epoxiconazole recorded a comparatively lesser activity against rice blast, while mancozeb + carbendazim was found to be least effective among all the fungicides in reducing leaf blast incidence. The highest disease control was achieved by azoxystrobin+ difenoconazole and azoxystrobin + tebuconazole, which were as effective as tricyclazole, and the least disease control was by mancozeb + carbendazim in both the years 2018 and 2019 (Figure 3a,b).

### 3.2. Effect of Fungicide Treatments on Leaf Blast Severity

The application of fungicides reduced the leaf blast severity in all the treatments as compared to the control in both the years. However, the chemicals varied in their activity against the disease. No statistically significant (*p* < 0.05) differences were observed between tricyclazole and combination fungicides azoxystrobin + difenoconazole, and azoxystrobin + tebuconazole in the years 2018 and 2019 (Table 2 and Table 3). Again, no statistically significant (*p* < 0.05) differences were observed between combination fungicide trifloxystrobin + tebuconazole and tricyclazole in the year 2019. The disease severities ± standard deviations recorded by azoxystrobin + difenoconazole, azoxystrobin + tebuconazole, and tricyclazole were 2.53 ± 0.55, 3.00 ± 0.20, and 2.95 ± 0.10, respectively, in the year 2018. This indicates that these combination fungicides were as effective as tricyclazole in reducing leaf blast severity in both the years 2018 and 2019. For the other two combination fungicides viz. trifloxystrobin + tebuconazole (in 2018) and tricyclazole + mancozeb (in both the years), although they significantly reduced the severity of leaf blast in comparison to the untreated control, the reduction in severity was significantly lower (*p* < 0.05) as compared to tricyclazole. The combination mancozeb + carbendazim recording a severity of 10.78 ± 0.58 was found to be least effective in reducing the leaf blast severity (Figure 3c,d).

### 3.3. Effect of Fungicide Treatments on Neck Blast Incidence

All the fungicidal treatments significantly reduced the incidence of neck blast as compared to the untreated control in both the years 2018 and 2019. The fungicide azoxystrobin + difenoconazole was significantly at par (*p* < 0.05) with tricyclazole in its activity against neck blast in the year 2018. All other fungicide treatments, although being effective in reducing neck blast incidence, showed slightly lesser activity as compared to tricyclazole (*p* < 0.05) in the year 2018 (Table 2). In the year 2019, two combination fungicides azoxystrobin + difenoconazole, and azoxystrobin + tebuconazole were recorded statistically at par (*p* > 0.05) with tricyclazole in their activity against neck blast (Table 3). All other fungicide treatments were found less effective than tricyclazole in reducing neck blast incidence. The least activity towards neck blast was again shown by mancozeb + carbendazim which recorded neck blast incidence of 38.31%. The highest disease control was achieved with tricyclazole, azoxystrobin+ difenoconazole, and azoxystrobin + tebuconazole and the least disease control with mancozeb + carbendazim in both the years 2018 and 2019 (Figure 3e,f).

### 3.4. Effect of Fungicide Treatments on Yield

The application of fungicides increased rice yields in all the fungicide treatments in both the years 2018 and 2019 (Table 4). All the treatments recorded significantly higher rice yields as compared to the untreated control. In the year 2018, the increase in yield due to the application of azoxystrobin + difenoconazole and azoxystrobin + tebuconazole was significantly at par (*p* < 0.05) with tricyclazole (Table 4). In the year 2019, although azoxystrobin + difenoconazole and azoxystrobin + tebuconazole recorded statistically lesser (*p* < 0.05) yields as compared to tricyclazole, the differences in yield were very small. The highest yields were recorded in treatments tricyclazole, azoxystrobin + difenoconazole, and azoxystrobin + tebuconazole, while all other fungicidal treatments recorded significantly lower yields than tricyclazole in both the years 2018 and 2019.

## 4. Discussion

Rice blast is the most destructive disease of cultivated rice in India. The efficacy of fungicides in controlling rice blast disease was investigated in the current study to determine the fungicides’ disease control potential under field conditions [40]. Azoxystrobin was reported to be more effective than propiconazole in controlling rice blast disease in the seedling stage in Australia [40]. New generation fungicides such as tricyclazole and propiconazole have been found to be highly effective in managing the disease under field conditions [41]. Tricyclazole + hexaconazole application has been reported to be most effective with thehighest percent of disease control of 87.08% and 79.62% for leaf and neck blast, respectively [42]. Tricyclazole was also reported as the fungicide with maximum efficiency, reducing leaf and neck blast by 89.2% and 97.5%, respectively, with a 43.3% increase in yield as compared to the control [43]. Tricyclazole was reported to be significantly superior against rice blast disease with the lowest PDI (16.01%) and highest percent of disease control [44]. In the current study, blast disease showed higher severity in 2019 compared to 2018 which may be due to higher rainfall in 2019 than 2018, as the disease has been reported to be positively correlated with the rainfall [45,46]. Tricyclazole was found to be the most effective fungicide in combating the blast disease under field conditions in both of the years. Tricyclazole exhibited the greatest efficacy with disease control of up to 89.43%, while the least disease control (58.66%) was recorded with the mancozeb + carbendazim application.

Tricyclazole prevents melanin biosynthesis in appressoria of *Pyricularia oryzae* and penetration of rice plants via appressoria by inhibiting either polyhydroxynapthaline reductase [25]. Tricyclazole inhibits the NADPH-dependent reduction of 1,3,6,8-tetrahydroxynaphthaline to scytalone and 1,3,8-trihydroxynaphthaline to vermelone [47]. The observations are supported by the work of the authors of [27], who reported a disease reduction of 67.90% with tricyclazole and the least reduction with mancozeb. The data presented (Table 2 and Table 3) reveal that the combination fungicides containing strobilurin and triazoles viz. azoxystrobin + difenoconazole (63.6%), and azoxystrobin + tebuconazole (62.4%) were almost as effective as tricyclazole in their activity against the rice blast disease. Trifloxystrobin + tebuconazole controlled the disease by (54.5%) and was found at par with tricyclazole + mancozeb.It was also recorded that azoxystrobin + difenoconazole (0.1%), and floxystrobin + tebuconazole (0.04%) were significantly effective against rice blast, recording a disease reduction of 55.1% and 53.3%, respectively [48]. Similar observations have been reported by other workers with triazole combination fungicides [27]. Some other workers have reported strobilurin fungicides to be more effective than tricyclazole for managing the rice blast disease [24,43]. In addition, strobilurin fungicides are reported to be active against grain discoloration, sheath rot, brown spots, and sheath blight of rice in addition to blast [49,50,51].

Other triazoles have also been reported to be efficient against rice blast. Blast reductions of 73–76% and 75–77% have been reported with tricyclazole and epoxiconazole, respectively [52]. Both of these fungicide groups, viz. strobilurins and triazoles, are single-site inhibitors. The triazole group inhibits sterol biosynthesis and strobilurins inhibit enzyme activities in mitochondrial respiration [53]. However, both of these fungicidal groups are designated as high-risk groups and the rice blast pathogen (*Pyricularia oryzae*) has been declared as a highly destructive plant pathogen by the Fungicide Resistance Action Committee [27]. Hence, these fungicides should be sprayed in rotation and used in combination with other groups possessing different mechanisms of action. In addition, they may be used in combination with low-risk fungicides which will help in the prevention of the accumulation of resistance in the pathogen populations. The application of fungicides resulted in increased rice yields in both the years (2018 and 2019) with the highest increase recorded in the case of tricyclazole treatments. Increased rice yields with tricyclazole application have also been reported by other workers [27,54]. The combinations of triazole and strobilurin fungicides were found to be highly effective against rice blast disease in fields with significant increases in yield. Hence, these combination fungicides can be used as alternatives to tricyclazole in rice blast management strategies.

## 5. Conclusions

*Mushk Budji* is a short bold aromatic landrace of rice in the temperate Himalayas of North India, with tremendous export potential because of its aroma and other quality attributes. Its huge susceptibility to blast disease is a constant challenge to its cultivation and potential adoption on a large scale by the rice farming community. The banning of tricyclazole in India has created a big gap in the fungicide management strategy against blast disease of rice. The current study has led us to the conclusion that combination fungicides with sterol biosynthesis + QoI inhibitors, succinate dehydrogenase + sterol biosynthesis inhibitors, and sterol biosynthesis + β-tubulin polymerization inhibitors could provide good management of rice blast disease under field conditions. The fungicides azoxystrobin + difenoconazole and azoxystrobin + tebuconazole were found to be as effective as tricyclazole in reducing the rice blast severity and increasing the rice yields. Hence, these combination fungicides can be used as alternatives to tricyclazole in rice blast management strategies.

## Figures and Tables

**Table 1:** Disease rating scale (IRRI, 2013).

Category	Symptoms
0	No lesion
1	Small brown specks of pinhead size without sporulating center
2	Small roundish to slightly elongated, necrotic grey spots, about 1–2 mm in diameter with a distinct brown margin and lesions are mostly found on the lower leaves
3	Lesion type is the same as in scale 2, but significant number of lesions are on the upper leaves
4	Typical sporulating blast lesions, 3 mm of longer, infecting less than 2% of the leaf area
5	Typical blast lesions infecting 2–10% of the leaf area
6	Blast lesions infecting 11–25% leaf area
7	Blast lesions infecting 26–50% leaf area
8	Blast lesions infecting 51–75% leaf area
9	More than 75% of leaf area affected

**Table 2:** Field efficacy of various fungicides on the severity of rice blast disease (2018).

Active Ingredient	Fungicide	Chemical Group	Concentration per Liter of Water	Leaf Blast Incidence	Neck Blast Incidence	Leaf Blast Severity
Flusilazole 12.5% + Carbendazim 25% SC	Lustre	triazole plus benzimidazoles	1 mL	21.88 ± 0.69 ^b^	35.27 ± 0.76 ^c^	7.45 ± 0.56 ^c^
Azoxystrobin 18.2% + difenconazole 11.4% SC	Amistar^®^ Top	triazole plus strobilurin	1 mL	8.97 ± 0.71 ^e^	12.56 ± 0.40 ^i^	2.53 ± 0.55 ^g^
Azoxystrobin 11% + tebuconazole 18.3% SC	Custodia	triazole plus strobilurin	1.5 mL	9.57 ± 0.57 ^e^	15.52 ± 1.25 ^h^	3.00 ± 0.20 ^f,g^
Tricyclazole 18% + mancozeb 62% WP	Merger	triazole plus dithiocarbamate	2.5 g	11.67 ± 0.64 ^d^	18.8 ± 0.54 ^g^	3.89 ± 0.35 ^e,f^
Zineb 68% + hexaconazole 4%	Avatar	triazole plus dithiocarbamate	2.5 g	17.09 ± 0.56 ^c^	28.12 ± 0.76 ^e^	6.21 ± 0.46 ^d^
Trifloxystrobin 25% + tebuconazole 50% WG	Nativo^®^	triazole plus strobilurin	0.4 g	11.54 ± 0.53 ^d^	20.19 ± 0.80 ^f^	4.89 ± 0.23 ^e^
Mancozeb 50% + Carbendazim 25% WS	Sprint^®^	dithiocarbamate plus benzimidazole	2.5 g	12.3 ± 0.96 ^d^	36.55 ± 0.68 ^b^	10.05 ± 1.07 ^b^
Fluxapyroxad 62.5 g/L + epoxiconazole 62.5 g/L EC	Adexar^®^	pyrazole-carboximide plus triazole	1.5 mL	22.57 ± 1.54 ^b^	30.83 ± 0.72 ^d^	7.61 ± 0.45 ^c^
Tricyclazole 75%	Force 11^TM^	melanin biosynthesis inhibitors—reductase	0.6 g	6.86 ± 0.90 ^f^	12.22 ± 0.35 ^i^	2.95 ± 0.10 ^f,g^
Water (Control)	-	-	-	64.92 ± 0.52 ^a^	87.50 ± 1.47 ^a^	26.24 ± 1.13 ^a^

Rice blast severity on a scale of 0–9, where 0 = no symptoms, and 9 = most severe (IRRI, 2013). Means ± standard deviations in each column followed by different superscripted letters are significantly different at *p* < 0.05 based on DMRT.

**Table 3:** Field efficacy of various fungicides on the severity of rice blast disease (2019).

Active Ingredient	Fungicide	Chemical Group	Concentration per Liter of Water	Leaf Blast Incidence	Neck Blast Incidence	Leaf Blast Severity
Flusilazole 12.5% + carbendazim 25% SC	Lustre	triazole plus benzimidazoles	1 mL	22.64 ± 0.94 ^c,b^	36.23 ± 1.50 ^b^	8.76 ± 0.80 ^c^
Azoxystrobin 18.2% + difenconazole 11.4% SC	Amistar^®^ Top	triazole plus strobilurin	1 mL	9.19 ± 1.35 ^f^	14.74 ± 2.21 ^f^	3.43 ± 0.46 ^f^
Azoxystrobin 11% + tebuconazole 18.3% SC	Custodia	triazole plus strobilurin	1.5 mL	10.40 ± 0.60 ^e,f^	16.64 ± 0.97 ^f^	3.82 ± 0.27 ^e,f^
Tricyclazole 18% + mancozeb 62% WP	Merger	triazole plus dithiocarbamate	2.5 g	12.49 ± 0.50 ^e^	20.01 ± 0.74 ^e^	4.93 ± 0.20 ^e^
Zineb 68% + hexaconazole 4%	Avatar	triazole plus dithiocarbamate	2.5 g	18.61 ± 1.11 ^d^	29.51 ± 1.34 ^d^	6.72 ± 0.42 ^d^
Trifloxystrobin 25% + tebuconazole 50% WG	Nativo^®^	triazole plus strobilurin	0.4 g	12.43 ± 0.56 ^e^	19.92 ± 0.86 ^e^	4.68 ± 0.87 ^e,f^
Mancozeb 50% + Carbendazim 25% WS	Sprint^®^	dithiocarbamate plus benzimidazole	2.5 g	27.61 ± 0.75 ^b^	38.31 ± 1.36 ^b^	10.78 ± 0.58 ^b^
Fluxapyroxad 62.5 g/L + epoxiconazole 62.5 g/L EC	Adexar^®^	pyrazole-carboximide plus triazole	1.5 mL	23.22 ± 0.39 ^c^	33.22 ± 0.39 ^c^	8.84 ± 0.76 ^c^
Tricyclazole 75%	Force 11^TM^	melanin biosynthesis inhibitors—reductase	0.6 g	8.41 ± 0.80 ^f^	14.10 ± 0.97 ^f^	3.56 ± 0.54 ^f^
Water (Control)	-	-	-	66.78 ± 7.04 ^a^	89.14 ± 4.22 ^a^	27.98 ± 1.72 ^a^

Rice blast severity on a scale of 0–9, where 0 = no symptoms, and 9 = most severe (IRRI, 2013). Means ± standard deviations in each column followed by different superscripted letters are significantly different at *p* < 0.05 based on DMRT.

**Table 4:** Effect of fungicide treatments on the yield of Mushk Budji rice in 2018 and 2019.

Active Ingredient	Fungicide	Chemical Group	Concentration Per Liter of Water	Yield (Quintal/Hectare) 2018	Yield (Quintal/Hectare) 2019
Flusilazole 12.5% + carbendazim 25% SC	Lustre	triazole plus benzimidazoles	1 mL	44.41 ± 3.53 ^d^	45.73 ± 1.88 ^e^
Azoxystrobin 18.2% + difenconazole 11.4% SC	Amistar^®^ Top	triazole plus strobilurin	1 mL	63.62 ± 3.20 ^a^	60.30 ± 2.21 ^b^
Azoxystrobin 11% + tebuconazole 18.3% SC	Custodia	triazole plus strobilurin	1.5 mL	62.43 ± 3.48 ^a^	60.29 ± 3.30 ^b^
Tricyclazole 18% + mancozeb 62% WP	Merger	triazole plus dithiocarbamate	2.5 g	56.09 ± 2.77 ^b^	54.23 ± 3.18 ^c^
Zineb 68% + hexaconazole 4%	Avatar	triazole plus dithiocarbamate	2.5 g	51.54 ± 3.35 ^c^	50.27 ± 2.31 ^d^
Trifloxystrobin 25% + tebuconazole 50% WG	Nativo^®^	triazole plus strobilurin	0.4 g	54.50 ± 2.94 ^b,c^	53.83 ± 2.73 ^c,d^
Mancozeb 50% + Carbendazim 25% WS	Sprint^®^	dithiocarbamate plus benzimidazole	2.5 g	41.80 ± 3.15 ^d^	38.96 ± 3.73 ^f^
Fluxapyroxad 62.5 g/L + epoxiconazole 62.5 g/L EC	Adexar^®^	pyrazole-carboximide plus triazole	1.5 mL	41.99 ± 3.53 ^d^	43.76 ± 2.80 ^e^
Tricyclazole 75%	Force 11^TM^	melanin biosynthesis inhibitors—reductase	0.6 g	65.85 ± 3.40 ^a^	67.73 ± 3.90 ^a^
Water (Control)	-	-	-	31.75 ± 2.50 ^e^	32.08 ± 3.27 ^g^

Means ± standard deviations in each column followed by different superscripted letters are significantly different at *p* < 0.05 based on DMRT.

## Data Availability

All data generated or analyzed in this study are available within the manuscript and are available from the corresponding authors upon request.
